# A pan-cancer analysis of the oncogenic role of polypyrimidine tract binding protein 1 (PTBP1) in human tumors

**DOI:** 10.1097/MD.0000000000032428

**Published:** 2022-12-30

**Authors:** Qing Huang, Shinong Gu, Jianqi Fang, Xuanwen Li, Lili Lin

**Affiliations:** a College of Environment and Public Health, Xiamen Huaxia University, Xiamen, Fujian, P.R. China; b Department of Women’s Health Care, Fujian Maternity and Child Health Hospital, Fuzhou, Fujian, P.R. China; c Graduate School of Health Science, Suzuka University of Medical Science, Suzuka, Mie, Japan.

**Keywords:** mutation, phosphorylation, PTBP1, survival analyses

## Abstract

Polypyrimidine tract-binding protein 1 (PTBP1) is an RNA-binding protein that regulates several posttranscriptional events and is closely related to the development of multiple tumors. However, little is known about PTBP1. Thus, we carried out a systematic pan-cancer analysis to explore the relationship between PTBP1 and cancer.

**Methods:**

We used The Cancer Genome Atlas, Gene Expression Omnibus, and Human Protein Atlas datasets, as well as several bioinformatics tools, to explore the role of PTBP1 in 33 tumor types.

**Results:**

The expression of PTBP1 in most tumor tissues was higher than that in normal tissues. Survival analysis indicated that overexpression of PTBP1 generally predicted poor overall survival in patients with tumors such as adrenocortical carcinoma, liver hepatocellular carcinoma, lung adenocarcinoma, and skin cutaneous melanoma. In addition, we compared the phosphorylation and immune infiltration of PTBP1 in cancer-associated fibroblasts between normal and primary tumor tissues and explored the putative functional mechanism of tumorigenesis mediated by PTBP1.

**Conclusion:**

These results provide clues to better understand PTBP1 from the perspective of bioinformatics and highlight its importance in various human cancers.

## 1. Introduction

Polypyrimidine tract-binding protein 1 (PTBP1) belongs to the subfamily of ubiquitously expressed heterogeneous nuclear ribonucleoproteins, the gene of which is located on chromosome 19p13.3 in humans.^[1]^ PTBP1 is a 57 kDa protein with an N-terminal nuclear shuttling domain and 4 RNA-binding domains of the RNA recognition motif (RRM) that to the polypyrimidine-rich region of the target RNA.^[2–6]^ PTBP1 belongs to the PTB family, which includes PTBP2 and PTBP3. PTBP1 is expressed in almost all cell types, PTBP2 is only expressed in the nervous system, and PTBP3 is mainly expressed in hematopoietic cells.^[7–10]^

PTBP1, a known regulator of posttranscriptional gene expression, is involved in alternative splicing and regulation of the polyadenylation efficiency of precursor mRNA, as well as mRNA stability; also, it is closely related to the development of multiple tumors.^[11–13]^ Previous studies have suggested that PTBP1 is highly expressed and participates in the malignant biological behavior of bladder, colon, and breast cancer cells.^[14–16]^ However, we have not yet reviewed any pan-cancer studies that focus on the relationship between PTBP1 and various tumor types. Therefore, we aimed to conduct a pan-cancer analysis of PTBP1 using the cancer genome atlas (TCGA) and Gene Expression Omnibus (GEO) databases. In addition, we investigated the potential molecular mechanism of PTBP1 by analyzing gene expression, survival status, genetic alterations, protein phosphorylation, immune infiltration, and relevant cellular pathways in various tumors.

## 2. Materials and Methods

### 2.1. Gene expression analysis

We entered PTBP1 into the “Gene_DE” module of the Tumor Immune Estimation Resource (version 2) (TIMER2) network (http://timer.cistrome.org/) and observed differences in the expression of PTBP1 between different tumors or specific tumor subtypes in TCGA. For some tumors with normal or highly normal tissue [for example, TCGA-glioblastoma multiforme (GBM) and TCGA-acute myeloid leukemia), we used the “Expression Analysis Box Plot” module of the Gene Expression Profiling Interactive Analysis (version 2) (GEPIA2) Network Server (http://gepia2.cancer-pku.cn/#analysis) to obtain box plot expression differences of these tumor and Genotype–Tissue Expression (GTEx) normal tissue databases, at a set *P*-value cutoff of .01, log_2_FC (fold change) cutoff of 1, and “Match TCGA normal and GTEx Data.” In addition, we obtained violin plots of PTBP1 expression in different pathological stages (stages I, II, III, and IV of all TCGA tumors) through the “Pathological Stage Map” module of HEPIA2. Expression data transformed from log2 [transcripts per million (TPM) + 1) were applied to the box or violin plots.

The UALCAN Portal (http://ualcan.path.uab.edu/analysis-prot.html) is an interactive network resource for analyzing cancer omics data, allowing us to perform a protein expression analysis of the clinical proteomics tumor analysis consortium (CPTAC) dataset. We investigated the expression levels of total and phosphorylated proteins in primary tumors and normal tissues at the S16, S53, T138, S140, S141, Y456, and S459 sites of PTBP1 (NM_031991). In addition, we used the input “PTBP1” to select the available datasets of 6 tumors, namely, breast cancer, ovarian cancer, colon cancer, clear cell renal cell carcinoma, uterine corpus endometrial carcinoma (UCEC), and lung adenocarcinoma (LUAD).

### 2.2. Survival prognosis analysis

We used the GEPIA2 “Survival Map” module to obtain explicit map data on the overall survival (OS) and disease-free survival (DFS) of PTBP1 in all TCGA tumors. Cutoff-high (50%) and cutoff-low (50%) values were used as the expression thresholds for splitting the high- and low-expression cohorts.^[17]^ The log-rank test was used as the hypothesis test, and survival plots were obtained using GEPIA2’s “survival analysis” module.

### 2.3. Genetic alteration analysis

After accessing the cBioPortalWeb (https://www.cbioportal.org/), we selected the “TCGA Pan-cancer Atlas Study” in the “Quick Selection” section and entered the “PTBP1” query for genetic alteration features of PTBP1. The frequency of changes, mutation type, and results of copy number alteration of all TCGA tumors were observed in the “Cancer Type Summary” module. We used a schematic diagram of the protein or 3D structure to exhibit the mutational site information of PTBP1 with the “mutational” module. We also obtained data using the “comparison” module on overall, disease-specific, disease-free, and progression-free survival differences in TCGA cancer cases with or without PTBP1 genetic alterations. Kaplan-Meier plots with log-rank *P* values were also generated.

### 2.4. Immune infiltration analysis

We used the “Immune-Gene” module of the TIMER2 Web server to explore the relationship between PTBP1 expression and immune infiltration in all TCGA tumors. T cells and tumor-associated fibroblasts were selected for further analyses. The TIMER, CIBERSORT, CIBERSORT-ABS, QUANTISEQ, XCELL, MCPCOUNTER, and EPIC algorithms were applied to estimate immune infiltration.^[18]^ *P*-values and partial correlation (cor) values were obtained using a purity-adjusted Spearman’s rank correlation test. The data were visualized as heat maps and scatter plots.^[19]^

### 2.5. PTBP1-related gene enrichment analysis

First, we searched the String website (https://string-db.org/) using a query of a single protein name (“PTBP1”) and an organism (“Homo sapiens”). Subsequently, we set the following main parameters: minimum required interaction score (“Low confidence [0.150]”), meaning of network edges (“evidence”), maximum number of interactions shown (“no more than 50 interactors” in 1^st^), and active interaction source (“experiments”). Finally, the experimentally determined PTBP1 binding protein was obtained.

To obtain the top 100 PTBP1-correlated genes, GEPIA2 was used based on all tumor and normal tissues from TCGA datasets. Then, a pairwise gene–gene Pearson correlation analysis was conducted between PTBP1 and the selected genes. The results of the analysis are indicated in the corresponding figure panels, including *P* values and the correlation coefficient (*R*). The heatmap representation of the expression profile for the selected genes contains the partial correlation (cor) and *P* value in the purity-adjusted Spearman’s rank correlation test.^[20]^

Kyoto Encyclopedia of Genes and Genomes (KEGG) pathway analysis was performed using 2 sets of data, and the “tidyr” and“ggplot2” R packages were used for the visualization of the enriched pathways. In addition, R language software [*R*-3.6.3, 64-bit] (https://www.r-project.org/) was used for this analysis. For all tests, a 2-tailed *P* < .05 was considered statistically significant.^[21]^

## 3. Results

### 3.1. Gene expression analysis data

**Figure 1.**
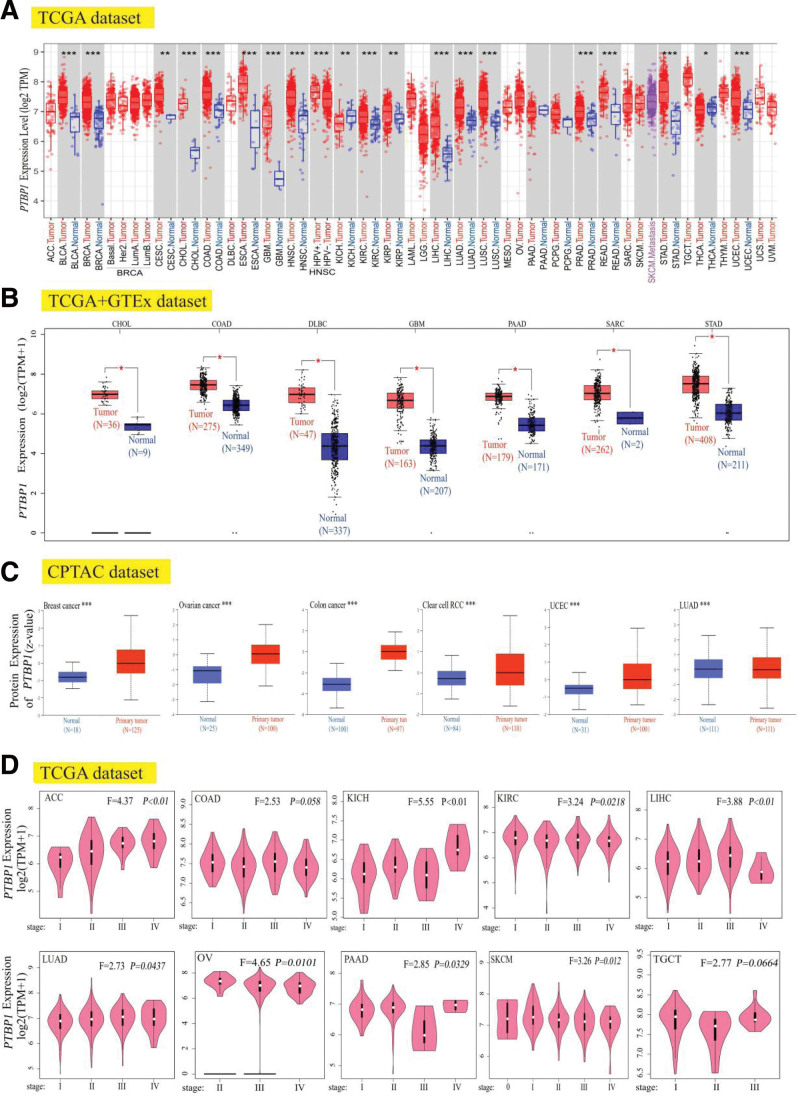
Expression level of PTBP1 gene in different tumors and pathological stages. (a) TIMER2 was used analyzed to exhibited the expression status of the PTBP1 gene in different cancers or specific cancer subtypes. * *P* < .05; ** *P* < .01; *** *P* < .001. (b)The box plot data were supplied for comparison of PTBP1 expression level in CHOL, COAD, DLBC, GBM, PAAD, SARC, and STAD between TCGA and GTEx database (corresponding normal tissues). * *P* < .05. (c) Based on the CPTAC dataset, the differences of the PTBP1 total protein expression level between normal tissue and primary tissue were analyzed, including breast cancer, ovarian cancer, colon cancer, clear cell RCC, UCEC, and LUAD. * *P* < .05; ** *P* < .01; *** *P* < .001. (d) The relationship between the PTBP1 expression levels and the main pathological stages (stage I, stage II, stage III, and stage IV) based on the TCGA data were analyzed, including ACC, COAD, KICH, KIRC, LIHC, LUAD, OV, PAAD, SKCM, and TGCT. Log2 (TPM + 1) was applied for log-scale. ACC = adrenocortical carcinoma, CHOL = cholangiocarcinoma, COAD = colon adenocarcinoma, KICH = kidney chromophobe, KIRC = kidney renal clear cell carcinoma, LIHC = liver hepatocellular carcinoma, LUAD = lung adenocarcinoma, OV = ovarian serous cystadenocarcinoma, PAAD = pancreatic adenocarcinoma, PTBP1 = polypyrimidine tract-binding protein 1, SARC = sarcoma, SKCM = skin cutaneous melanoma, STAD = stomach adenocarcinoma, TCGA = the cancer genome atlas, TGCT = testicular germ cell tumor.

We integrated tumor and normal samples from TCGA databases to identify PTBP1 mRNA expression characteristics. As shown in Figure 1a, the expression level of PTBP1 in the tumor tissues of bladder urothelial carcinoma, breast invasive carcinoma, cholangiocarcinoma (CHOL), colon adenocarcinoma (COAD), esophageal carcinoma, GBM, head and neck squamous cell carcinoma, kidney renal clear cell carcinoma (KIRC), liver hepatocellular carcinoma (LIHC), LUAD, lung squamous cell carcinoma, prostate adenocarcinoma, rectum adenocarcinoma, stomach adenocarcinoma (STAD), UCEC (*P* < .001), cervical squamous cell carcinoma and endocervical adenocarcinoma, kidney renal papillary cell carcinoma (KIRP) (*P* < .01), and thyroid carcinoma (*P* < .05) was higher than that of normal tissues, while the expression level of PTBP1 in the tumor tissues of kidney chromophobe (KICH) was lower than that in normal tissues.

After including the normal tissues of the GTEx dataset as controls, we further evaluated the difference in PTBP1 expression between normal and tumor tissues. We found that CHOL, COAD, lymphoid neoplasm diffuse large B-cell lymphoma, GBM, pancreatic adenocarcinoma (PAAD), sarcoma (SARC), and STAD showed higher expression in the tumor tissues (Fig. 1b, *P* < .05).

The results of the CPTAC dataset showed higher expression of PTBP1 total protein in LUAD, COAD, ovarian cancer, clear cell renal cell carcinoma, breast cancer, and UCEC tissues than in normal tissues (Fig. 1c, *P* < .001).

In addition, the “Pathological Stage Plot” module of HEPIA2 was used to investigate the relationship between the expression levels of PTPB1 and the main pathological stages of cancers such as adrenocortical carcinoma (ACC), KICH, KIRC, LIHC, LUAD, ovarian serous cystadenocarcinoma, PAAD, and skin cutaneous melanoma (SKCM) (Fig. 1d, all *P* < .05).

### 3.2. Survival analysis data

**Figure 2.**
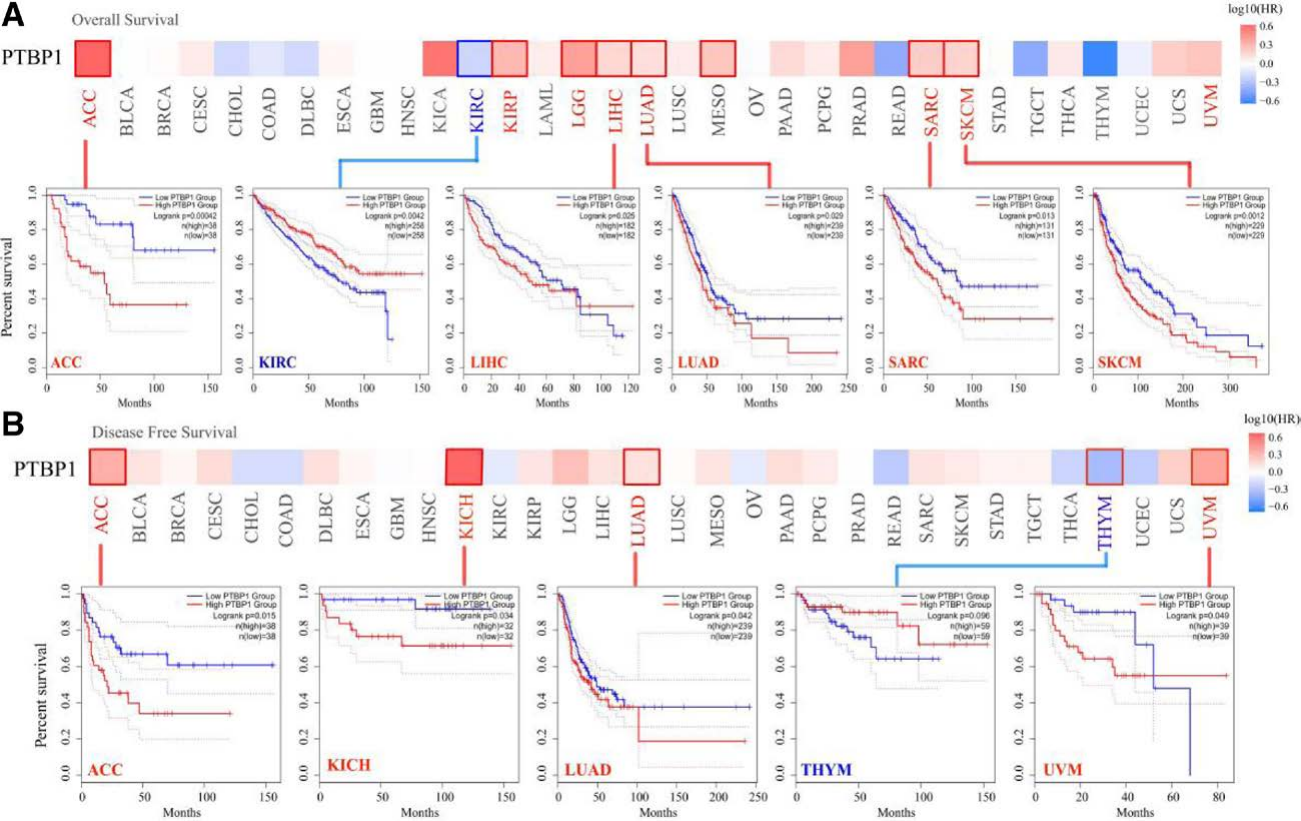
Correlation between PTBP1 gene expression and survival prognosis of cancers in TCGA. Survival analyzed was conducted using the GEPIA2 tool and exhibited as survival map and Kaplan-Meier curves. (a) Overall survival. (b) Disease-free survival. PTBP1 = polypyrimidine tract-binding protein 1, TCGA = the cancer genome atlas.

We used TCGA and GEO datasets to explore the relationship between the expression levels of PTBP1 and the prognosis of patients with various tumors. The cancer cases were divided into 2 groups based on the expression levels of PTBP1. As shown in Figure 2a, highly expressed PTBP1 negatively impacted the prognosis of OS, such as in the cases of ACC (*P* < .001), LIHC (*P* = .025), LUAD (*P* = .029), SARC (*P* = .013), and SKCM (*P* = .0012). However, low expression of the PTBP1 gene was related to poor OS prognosis for KIRC (*P* = .0042). The DFS analysis data in Figure 2b show a correlation between high PTBP1 expression and poor prognosis in TCGA cases of ACC (*P* = .015), KICH (*P* = .034), LUAD (*P* = .042), and uveal melanoma (*P* = .049).

### 3.3. Genetic alteration analysis data

**Figure 3.**
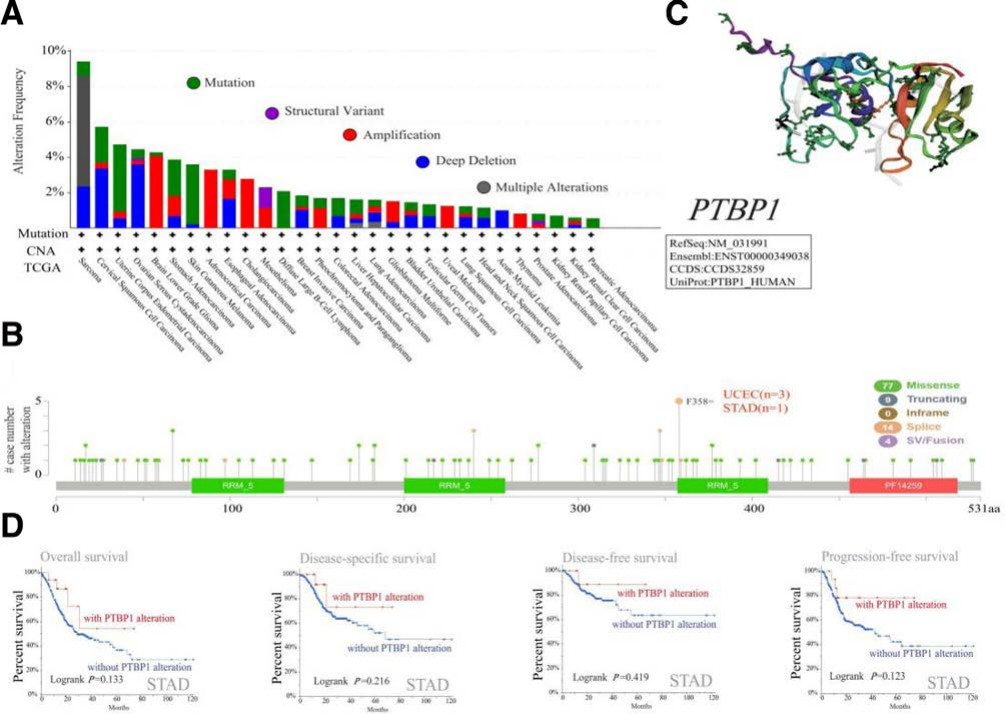
Mutation feature of PTBP1 in different tumors of TCGA. Using the cBioPortal tool, the alteration frequency with mutation type (a) and mutation site (b) are displayed. 3D structure of PTBP1 exhibited the mutation site with the highest alteration frequency (f358). (c). Also by the cBioPortal tool, we analyzed the correlation between mutation status and overall, disease-specific, disease-free and progression-free survival of STAD (d). PTBP1 = polypyrimidine tract-binding protein 1, STAD = stomach adenocarcinoma, TCGA = the cancer genome atlas.

Next, genetic alteration analysis of PTBP1 was conducted based on TCGA datasets. As shown in Figure 3a, the mutation had the highest alteration frequency of PTBP1 (~9%), which appeared in patients with SARC tumors. Furthermore, the “amplification” alteration type of copy number alteration was the main type in the brain lower grade glioma cancer cases, which showed an alteration frequency of ~4% (Fig. 3a). Additionally, the ACC, CHOL, uveal melanoma, and THYM cases with genetic alterations showed amplification of PTBP1, whereas all diffuse large B-cell lymphoma, KIRP, and PAAD cases with genetic alterations showed mutations in PTBP1 (Fig. 3a). Figure 3b further demonstrates the type, site, and number of cases of genetic alterations in PTBP1. The frequency of missense mutations in PTBP1 was higher than that of other types of genetic alterations. Moreover, F358 alteration in the RRM5 domain could induce a splice mutation in the PTBP1 gene, which was detected in 3 cases of UCEC and 1 case of STAD. Figure 3c shows the 3D structure of PTBP1. Furthermore, no association was found between genetic alterations of PTBP1 and the clinical survival prognosis of patients with all types of cancer in this study. The outcomes of STAD are shown as an example in Figure 3d.

### 3.4. Protein phosphorylation analysis data

**Figure 4.**
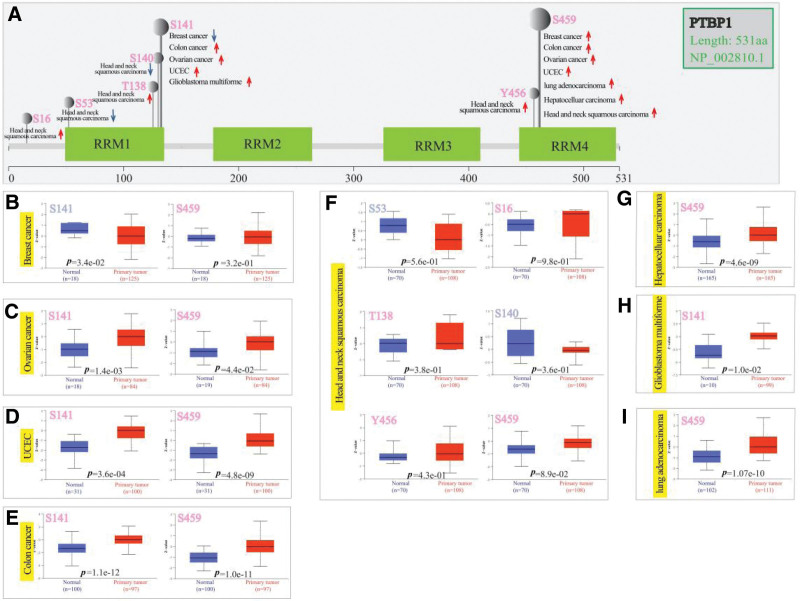
Phosphorylation analysis of PTBP1 protein in various tumors. Based on the CPTAC dataset, the differences of PTBP1 phosphoprotein (NP_002810.1, S16, S53, T138, S140, S141, Y456, and S459 sites) between normal tissue and of selected primary tumor tissue was analyzed via the UALCAN. The schematic diagram of PTBP1 protein exhibited positive results of phosphoprotein sites (a). We also supply the box plots for different cancers, including breast cancer (b), ovarian cancer (c), UCEC (d), colon cancer (e), HNSC (f), HCC (g), GBM (h), and LUAD (i). CPTAC = clinical proteomics tumor analysis consortium, GBM = glioblastoma multiforme, HCC = hepatocellular carcinoma, LUAD = lung adenocarcinoma, PTBP1 = polypyrimidine tract-binding protein 1, UCEC = uterine corpus endometrial carcinoma.

Seven types of tumors (ovarian cancer, breast cancer, colon cancer, UCEC, and LUAD) were analyzed based on the CPTAC dataset to compare the phosphorylation levels of PTBP1 in tumor and normal tissues. The PTBP1 phosphorylation sites and their significant differences are shown in Figure 4a. The phosphorylation level of the S459 locus within the RRM4 domain of PTBP1 was higher than that in normal tissues in almost all primary tumor tissues except GBM (Fig. 4a–g, i, all *P* < .05), followed by the S141 locus within the RRM1 domain for colon cancer, ovarian cancer, UCEC, and GBM (Fig. 4a, c–e, h, all *P* < .05), which also exhibited increased phosphorylation levels. In contrast, the S141 locus showed a decreased phosphorylation level in breast cancer cells (Fig. 4a, b, *P* < .05).

### 3.5. Immune infiltration analysis data

**Figure 5.**
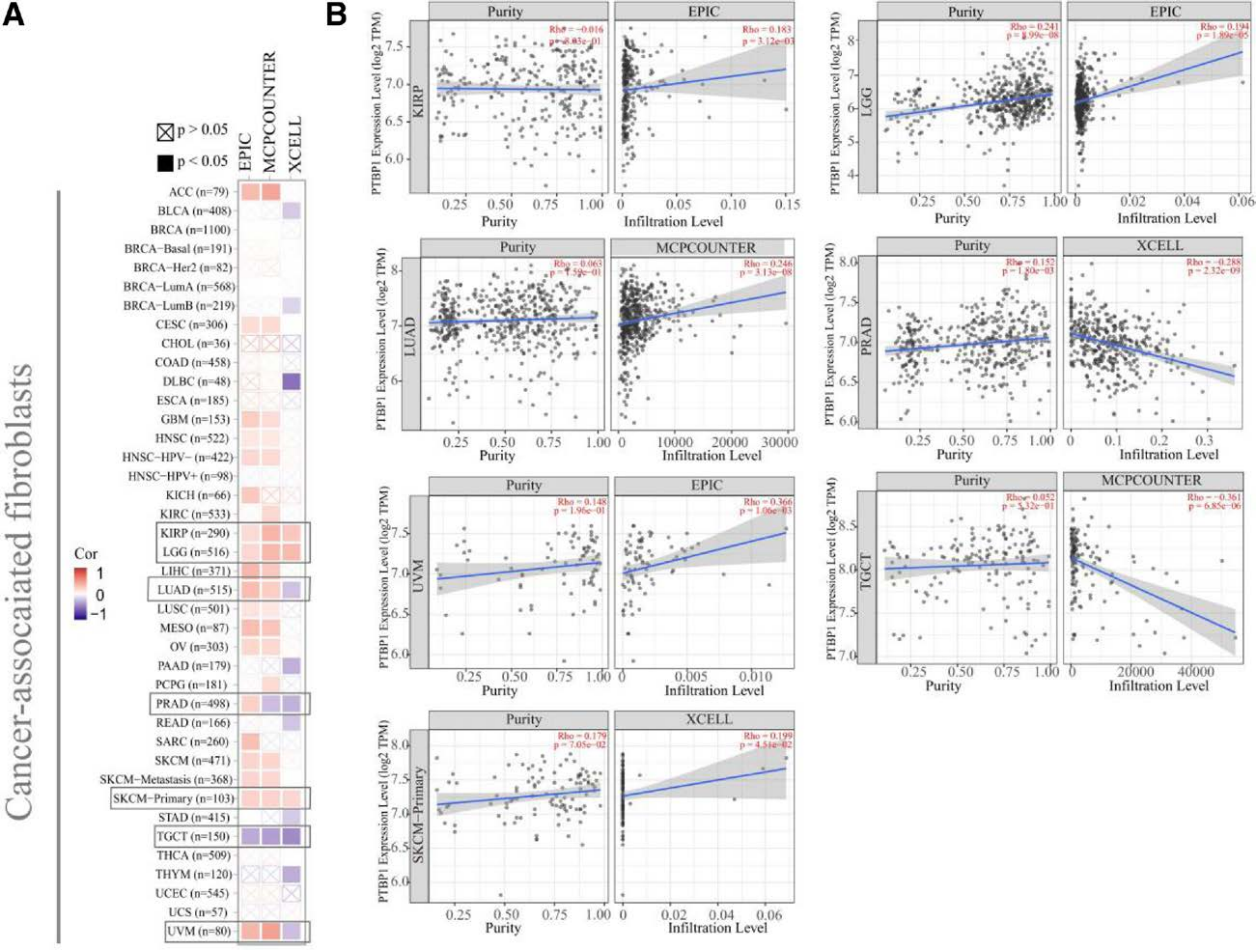
Correlation analysis between PTBP1 expression and immune infiltration of cancer-associated fibroblasts. We used different algorithms to explore the potential correlation between PTBP1 expression level and the infiltration level of cancer-associated fibroblasts across all types of cancer in TCGA. PTBP1 = polypyrimidine tract-binding protein 1, TCGA = the cancer genome atlas.

As shown in Figure 5, PTBP1 expression was statistically positively correlated with the estimated infiltration value of cancer-associated fibroblasts for TCGA tumors of SKCM, KIRP, and lower grade glioma, but negatively for testicular germ cell tumors. Figure 5 also displays the scatterplot data of these tumors produced using 1 algorithm. For example, using the MCPCOUNTER algorithm, we found a negative linear relationship between the expression level of PTPB1 in testicular germ cell tumors and the infiltration level of cancer-associated fibroblasts (Fig. 5, Rho = −0.361, *P* = 6.85e-06).

### 3.6. Enrichment analysis of PTBP1-related partners

**Figure 6.**
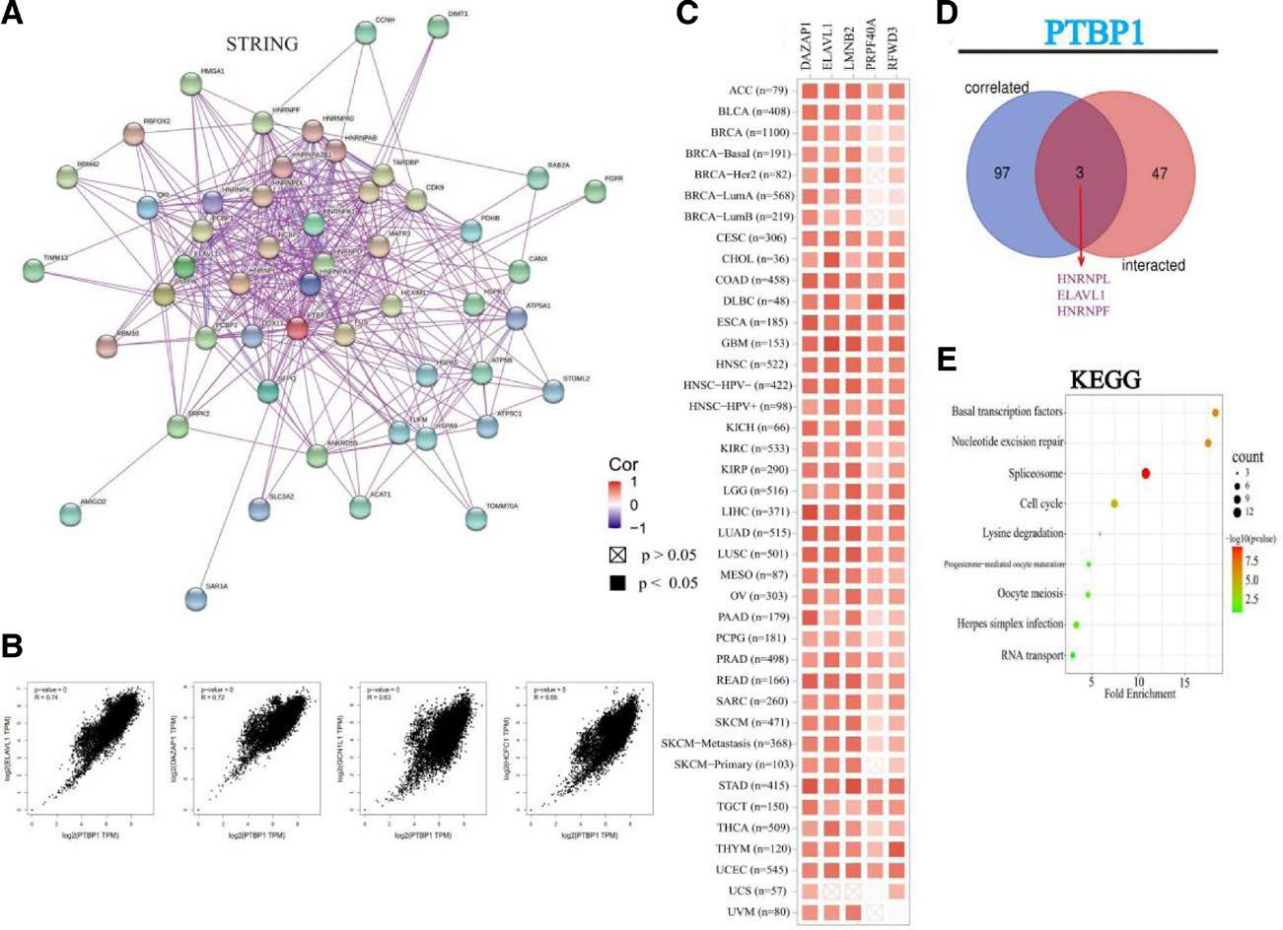
PTBP1-related gene enrichment analysis. (a) Based on the STRING tool, we obtained the available experimentally determined PTBP1-binding proteins. (b) Based on the GEPIA2 approach, the top 100 PTBP1-correlated genes in TCGA projects were obtained and the expression correlation between PTBP1 and selected targeting genes were analyzed, including ELAVL1, DAZAP1, GCN1L1, and HCFC1. (c) Heatmap data in the detailed cancer types are displayed. (d) Intersection between the PTBP1-binding and correlated genes. (e) KEGG pathway analysis was performed based on the PTBP1-binding and interacted genes. ELAVL1 = embryonic lethal abnormal vision-like 1, DAZAP1 = deleted in azoospermia-associated protein 1, GCN1L1 = general control of amino-acid synthesis 1-like 1, KEGG = Kyoto encyclopedia of genes and genomes, PTBP1 = polypyrimidine tract-binding protein 1, TCGA = the cancer genome atlas.

Finally, we screened out genes targeting PTPB1-binding proteins and related genes for a series of pathway enrichment analyses to further study the molecular mechanism of the PTPB1 gene in tumorigenesis. Using the STRING tool, we obtained 50 PTBP1-binding proteins supported by experimental evidence. The interaction network of these 50 proteins is shown in Figure 6a. We used the GEPIA2 tool to combine all tumor expression data from TCGA and acquired the top 100 genes that were correlated with the expression of PTBP1. The expression of PTPB1 was positively associated with that of embryonic lethal abnormal vision-like 1 (*R* = 0.74), azoospermia-associated protein1 (*R* = 0.72), general control of amino-acid synthesis 1 like 1 (*R* = 0.63), and host cell factor C1 (*R* = 0.65) (Fig. 6b). We found similar results in the heatmap data for most cancer types, with PTPB1 having a strong positive correlation with the above 5 genes (Fig. 6c). Intersection analysis of the above 2 groups showed 1 common member, HNRNPELAVL1HNRNPF (Fig. 6d). We also combined the 2 datasets to perform KEGG enrichment analyses. The KEGG data in Figure 6e suggest that “splicesome” might be involved in the effect of PTBP1 on tumor pathogenesis.

## 4. Discussion

PTBP1 is a shuttle protein that moves between the nucleus and cytoplasm.^[22]^ In the nucleus, PTBP1 performs functions associated with alternative splicing and polyadenylation, whereas in the cytoplasm, it is involved in mRNA localization, stability, and translation.^[23]^ In cancer, PTBP1 is primarily involved in glycolysis, apoptosis, proliferation, tumorigenesis, invasion, and migration.^[1]^ We found no pan-cancer studies of PTBP1 through our literature search. Therefore, we searched the TCGA, CPTAC, and GEO databases to examine PTBP1 genes in 33 different tumors.

In our study, PTBP1 was overexpressed in the majority of tumor tissues compared to that in normal tissues. However, we obtained different conclusions for different tumors through the survival prognostic analysis of the PTBP1 gene. The results showed that high expression of PTBP1 in patients with ACC was associated with poor OS prognosis (*P* = .029), poor DFS (*P* = .042), and pathological stages (*P* < .01). However, the role of PTBP1 in ACC tumors has rarely been reported. These results may provide a new clinical biomarker for predicting the survival of patients with ACC.

Regarding lung cancer, we found a correlation between high expression of PTBP1 and poor OS prognosis (*P* = .029) and poor DFS (*P = *.042) specific for LUAD but not for lung squamous cell carcinoma. Nevertheless, the current study points to an inverse association between the expression level of PTBP1 and all types of lung tumors. Wu et al (2021) reported that the positive feedback loop of circGLIS3/miR-644a/PTBP1 promotes the malignant progression of non-small cell lung cancer.^[24]^ Similarly, according to Li et al (2019), PTBP1 enhanced exon11a skipping in a human ortholog of mammalian enabled pre-mRNA, which promoted migration and invasion in lung carcinoma cells.^[25]^ Further research should be conducted to explore the potential role of PTBP1 in the tumorigenesis of lung tumors.

Based on our analysis, high expression of PTBP1 is associated with poor OS in patients with LIHC. Kang et al (2019) found that inhibition of PTBP1 expression reduced cyclin D3 levels and hepatocellular carcinoma (HCC) cell growth.^[26]^ Shen et al (2020) indicated that PTBP1 affects the invasion and metastasis of HCC cells by regulating the alternative splicing of Axl exon 10.^[27]^ Another study showed that small nucleolar RNA host gene 6 promoted HCC progression via mRNA attenuation in the SET domain containing 7 and leucine zipper transcription factor-like 1 by acting as a decoy plus guide for heterogeneous nuclear ribonucleoprotein L and PTBP1.^[28]^ These results indicate that PTBP1 plays a vital role in the development of LIHC.

Our TCGA-based survival analysis results also indicated a correlation between high expression of PTBP1 and poor OS, as well as immune infiltration of cancer-associated fibroblasts. Marzese et al (2015) reported that PTBP1 knockdown significantly decreased the expression of CD44 splicing variant 6, thus reducing melanoma brain metastases.^[29]^

We also explored the molecular mechanism of the total protein and phosphoproteins of PTBP1 proteins in breast cancer, colon cancer, ovarian cancer, and UCEC using the CPTAC dataset. The results of this study indicated high expression of PTBP1 total protein and phosphorylation at S459 within the RRM4 domain in primary tumors compared with normal controls. However, the expression level of PTBP1 was not significantly associated with the overall survival of these patients. We still cannot exclude the possibility that high PTBP1 phosphorylation of S459 is a byproduct of dysregulated signaling with no functional significance in tumor cells.

## 5. Conclusions

In conclusion, our first pan-cancer analysis of PTBP1 demonstrated a statistical correlation between the expression of PTBP1 and clinical prognosis, protein phosphorylation, immune cell infiltration, tumor mutation burden, and microsatellite instability across multiple tumors, contributing to the elucidation of the role of PTBP1 in tumorigenesis from multiple perspectives.

## Author contributions

**Conceptualization:** Qing Huang, Shinong Gu, Jianqi Fang.

**Data curation:** Xuanwen Li.

**Formal analysis:** Qing Huang, Shinong Gu, Jianqi Fang.

**Funding acquisition:** Lili Lin.

**Methodology:** Xuanwen Li.

**Supervision:** Lili Lin.

**Writing – original draft:** Qing Huang, Shinong Gu, Jianqi Fang.

**Writing – review & editing:** Lili Lin.
